# Trajectory Analysis of Glycemic Control in Adolescents with Type 1 Diabetes Mellitus at Dammam Medical Complex, Saudi Arabia

**DOI:** 10.1155/2020/1247294

**Published:** 2020-12-22

**Authors:** Sherifa A. Alsada, Ebtesam M. Ba-Essa, Alya A. Alsaffar

**Affiliations:** ^1^Department of Family Medicine, Safwa General Hospital, Ministry of Health, Safwa, Eastern Province, Saudi Arabia; ^2^Department of Endocrinology, Diabetes and Endocrine Center, Dammam Medical Complex, Ministry of Health, Dammam, Eastern province, Saudi Arabia

## Abstract

**Background:**

Saudi Arabia is reported to have the highest number of children and adolescents with T1DM. However, data concerning glycemic control during adolescence are lacking.

**Objectives:**

To determine glycemic control at transition stage from pediatric to adult clinics, determine HBA1c patterns during follow-up, and identify any clinical or demographic variables that may predict a distinctive glycemic pattern.

**Design:**

Observational retrospective study. *Setting*. Dammam Medical Complex, secondary care hospital. *Patients and Method*. Adolescents aged ≥12 years, with HbA1c recorded at least once a year over 4 years of follow-up, were eligible for inclusion. A trajectory analysis from 2008 to 2019 was conducted, using latent class growth modelling (LCGM), and two-sample *t*-tests and Fisher's exact tests were conducted to determine whether there was a statistically significant difference in demographic and clinical variables. *Sample Size*. 44 patients.

**Results:**

61.36% were referred from pediatric clinics, and 84% were on multiple insulin daily injections. For the trajectory prediction, two groups were identified. Group 1 comprised 71.7%, had high HbA1c values at age 13 (HbA1c, 11.28%), and had a significant and stable decrease in HbA1c values with age (−0.32, *p* < 0.00). Group 2 comprised 28.2%, showed poor HbA1c values at age 13 (HbA1c, 13.28%), and showed increase in HbA1c values slightly by age 15, which then steadily decreased with age (−0.27). Results indicated that the initial HBA1c value was a significant predictor for group trajectory (*p*=0.01), while the remaining variables did not have any significance.

**Conclusion:**

Our study identified two groups with poorly controlled diabetes; however, the first group performed relatively better than the second group. Both groups almost doubled their targets, with a trend towards HbA1c reduction by the age of 19 in both groups. *Limitations*. Retrospective study with convenient, small sample size.

## 1. Introduction

Type 1 diabetes mellitus (T1DM) is characterized by absolute insulin deficiency [1]. According to the 2019 International Diabetes Federation (IDF) Atlas of the Middle East and North Africa, Saudi Arabia is one of the countries with highest age-adjusted comparative diabetes prevalence rates, reaching 18.3%, and with the highest number of children and adolescents with T1DM (age range, 0–19 years) (*n* = 27,800). Moreover, Saudi Arabia has been reported to have the highest number of new cases of T1DM in children and adolescents (*n* = 3,700) [2].

Glycated hemoglobin (HbA1c) test values are currently the only long-term glycemic control measure with robust outcome data. Follow-up data from the Diabetes Control and Complications Trial (DCCT) indicated that improved glycemic control between 5 and 7 years, including during adolescence and young adulthood, resulted in a decreased risk of microvascular and macrovascular complications and mortality in subsequent years [3].

The change from adolescence to adulthood is a period of major transition that involves increasing independence, a seeking of peer acceptance, and greater body image importance, along with increased insulin requirements due to increased insulin resistance. These changes often can lead to decreased adherence to regular blood glucose monitoring and insulin administration, which is reflected in terms of glycemic control, leading to increased morbidity and early mortality [4–7].

However, despite the increase in the incidence and prevalence of T1DM in Saudi Arabia, data concerning glycemic control during adolescence are lacking [8]. Furthermore, identifying predictors of worsening glycemic control is important to implement strategies that aim to prevent deterioration in glycemic control.

The Dammam Medical Complex is the only general adult governmental hospital serving the Dammam area in the eastern province of Saudi Arabia, and referrals are mainly derived from the Maternity and Children's Hospital (MCH) in Dammam. Due to cultural considerations, the transfer of care for patients from pediatric to adult clinics is undertaken between the ages of 12 and 14 years, instead of 18 years, as in most developed countries.

This study aimed the following:

1. Determine glycemic control at the transition stage from pediatric to adult clinics
2. Compare the level of control between newly diagnosed patients and patients referred from the pediatrics clinic
3. Determine if HbA1c levels in early adolescence are predictive of HbA1c levels in early adulthood
4. Determine HbA1c patterns during follow-up
5. Identify any clinical or demographic variables that may predict a distinctive glycemic pattern

The analysis was approved by the local ethical committee number RAC 058.

## 2. Methods

In this observational, retrospective study, from 2008 to 2019, we used trajectory analysis to identify any distinctive patterns of change in HbA1c values over several years of follow-up for each patient (either referred or newly diagnosed) from the age of presentation until 19 years of age.

For this retrospective analysis, an index group was identified using an electronic health information system and paper files review analysis of 44 patients was conducted, either new referrals of adolescents with T1DM to the adult endocrinology clinic or the newly diagnosed patients who started follow-up after presenting to the emergency department with diabetic ketoacidosis in Dammam medical complex.

Both males and females, aged ≥12 years, with HbA1c data recorded at least once a year over 4 years of follow-up were eligible for inclusion. Data were collected using data abstraction sheet for each patient (Supplement Table 1). Patients with fully completed data sheets were included in the analysis as a convenience sample.

Latent class growth modelling (LCGM) [9] was used to identify specific subgroups of individuals and to identify any distinctive patterns of change over time concerning glycemic control (HbA1c values). The SAS procedure “PROC TRAG,” developed by Jones, Nagin, and Roeder, in 2001, was implemented using SAS version 9.4 (SAS Inc., Cary, NC) to identify trajectories of glycemic control.

In this study, we adopted the procedures established by Nagin [9] that have previously been implemented in several other studies [10–14] to identify the number of groups representing relatively homogenous clusters of trajectories of HbA1c over the study period. This involved (1) determining the number of groups; (2) selecting the shape of the patterns of change for each group over time; and (3) determining model adequacy for the final model. Detailed statistical analyses are presented in the appendices (Supplement 2).

After selecting the best model, based on indicators of model fit and classification accuracy (a two-group model was identified), two-sample *t*-tests and Fisher's exact tests were conducted to determine whether there was a statistically significant difference in demographic and clinical variables, which included the following:

1. Initial age
2. Age at first diagnosis of T1DM
3. Initial body mass index (BMI)
4. Initial HbA1c
5. Patient type (two levels: emergency department (ED) (patients newly diagnosed) vs. MCH (patients referred from the pediatrics clinic))
6. Insulin delivery method (multiple daily injections (MDI) vs. mixed forms (MIX))
7. Sex
8. The average number of office visits
9. History of diabetic ketoacidosis (DKA) not including an initial presentation of T1DM diagnosis in the ED
10. Hypothyroidism
11. Dyslipidemia

Finally, a logistic regression was conducted to determine if any of the demographic and clinical variables could predict the trajectories. The results are shown as odds ratios (ORs) with 95% confidence intervals (CIs). For all statistical analysis, a *p* value <0.05 indicated statistical significance.

## 3. Results

### 3.1. Patient Demographic Characteristics

Data concerning 44 patients with T1DM were collected between 2008 and 2019 and analyzed in this study. Over the course of the years, starting as young as 12 years of age and ending as old as 19 years, patients were observed 4–8 times, which resulted in a total number of 272 observations for the study. Tables 1 and 2 present the baseline demographics of the patients participating in this study. Among the 44 study patients, nearly two-thirds (61.36%) were referred from the pediatrics clinic. All patients were Saudis with 54.55% being female. Most patients had started treatment for T1DM using separate multiple daily injections (MDI, 84.09%). Concerning comorbidities, a considerable number of patients had an episode of DKA (34.09%), dyslipidemia (20.45%), and hypothyroidism (6.82%), with one patient with retinopathy and 3 patients had sickle cell trait (SCT).

### 3.2. Trajectory Analysis

**Figure 1:**
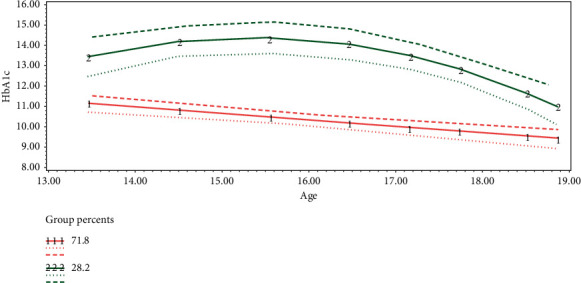
Longitudinal trajectories of HbA1c across adolescence (dash lines are 95% CIs).

Considering the small sample size, the visual inspection of the data, and the results of model selection based on Bayesian information criterion (BIC), a 2-group solution was selected for this study (Figure 1).

The first latent class (Group 1) comprised 71.76% (*n* = 32) of the sample, had high HbA1c values at age 13 (HbA1c, 11.28%), and had a significant and stable decrease in HbA1c values with age (−0.32, *p* < 0.00). The second latent class (Group 2) comprised 28.24% (*n* = 12) of the sample. Group 2 showed poor HbA1c values at age 13 (HbA1c, 13.28%) and these HbA1c values had increased slightly by age 15 and then steadily decreased with age thereafter (−0.27). Group 1 comprises 71.8% of the patients, and Group 2 comprises 28.2% of the patients.

### 3.3. Demographics and Trajectories

Tables 3 and 4 present demographic characteristics according to trajectory groups. According to two-sample *t*-tests, the initial BMI was statistically significantly higher for patients in Group 1 than for patients in Group 2 (mean: 22.6, SD 3.9 for Group 1; mean: 19.2, SD 3.0 for Group 2; *t* (41) = 2.6, *p*=0.01). The initial HbA1c values were statistically significantly higher for patients in Group 2 than for patients in Group 1 (mean: 10.9, SD 1.9 for Group 1; mean: 13.2, SD 2.3 for Group 2; *t* (42) = −3.30, *p*=0.00). There was no statistically significant difference in initial age (*p*=0.10), average number of office visits (*p*=0.10), and age at first diagnosis of T1DM (*p*=0.40), between Groups 1 and 2.

Fisher's exact test results showed no statistically significant association between the trajectory group and patient type (*p*=1.0), insulin regimen (*p*=0.3), gender (*p*=0.7), DKA (*p*=1.0), hypothyroidism (*p*=1.0), and dyslipidemia (*p*=0.08).

### 3.4. Trajectory Prediction

A logistic regression analysis was conducted to determine if any of the demographic and clinical variables could predict the trajectories. The results indicated that the initial HbA1c value was a significant predictor for group trajectory (*p*=0.01). In particular, patients with a higher initial HbA1c value were 98.47% less likely to be in Group 1 (OR 0.01; 95% CI, 0.31–0.88). The remaining variables, including patient type (*p*=0.17), initial insulin regimen (*p*=0.33), gender (*p*=0.73), history of DKA (*p*=0.74), hypothyroidism (*p*=0.77), dyslipidemia (*p*=0.13), initial age (*p*=0.30), initial BMI (*p*=0.12), average number of office visits (*p*=0.38), and age of first diagnosis of T1DM (*p*=0.56), were not significant predictors for group trajectory.

## 4. Discussion

This novel study, involving 2 groups, evaluated the trajectory of glycemic control in adolescents with T1DM in Saudi Arabia. The first group, with an average HbA1c value of 11.28%, showed a steady decrease of 0.34% by the age of 19. The second group showed worse glycemic control, with an average HbA1c value of 13.28% that increased until the age of 15 years and then showed a steady decrease of 0.27% by the end of analysis period, and the initial HbA1c value was the statistically significant predictor of the group. Although the initial BMI was statistically significantly higher for patients in Group 1 than for patients in Group 2, its clinical implication cannot be determined as it is not the proper method for assessing obesity at this age group.

Internationally, there have been a limited number of studies that have evaluated glycemic control trajectories for patients with T1DM [11, 15–21], and many studies have focused on the psychiatric aspects of T1DM and the family dynamic effects concerning glycemic control for adolescents with T1DM [15, 16]. All identified studies have been prospective except for one retrospective study, and the duration of follow-up ranged between 3 years and 11 years. The number of patients involved ranged from 132 to 6433 patients, and between 2 and 5 patterns were identified ranging between stable well-controlled groups and deteriorating poorly controlled groups. Variables such as gender, age at diagnosis, and duration of diabetes did not differ among participants in any of the groups; however, the use of an insulin pump and self-monitoring of blood glucose were found to be more frequent among participants in the stable groups. Only one study reported that girls were more likely to be in the poorly controlled groups [18].

Compared to other studies, our findings did not identify a group of relatively well-controlled patients or those who sustained stable control over time, and both clinical and demographic variables did not significantly predict group membership. Moreover, there was no difference found between patients who had been referred from the pediatrics clinic and the patients who started their follow-up at the Centre from the time of initial diagnosis. These results indicated that the transition of service from the pediatrics department to an adult clinic did not affect glycemic control, contrary to what we had expected, given there is no coordination between the two hospitals, and because studies focusing on this critical crossover period have reported a deterioration in glycemic control during this crossover phase for patients. Our findings might be explained due to the retrospective nature of the study, the small sample size, and the fact that no patient was on continuous subcutaneous insulin infusion therapy, which had a significant effect on being a member of a good control group in other studies. Despite the limitations of our sample, these results highlight a major health issue with considerable future implications for patients and for the large number of adolescent patients with T1DM in Dammam, Saudi Arabia. We identified two groups with poorly controlled diabetes; however, the first group performed relatively better than the second group. Both groups almost doubled their targets, with a trend towards HbA1c reduction by the age of 19 in both groups; however, it is not clear if this reduction would be likely to continue in subsequent years, or even if this trend was to persist, DCCT study results suggest that the health consequences in terms of glycemic derangement would be likely to persist beyond this period [7].

## Data Availability

The data used in the study are available upon request to the corresponding author.

## Supplementary Materials

Supplementary Materials Table 1: patient demographic data (categorical variables). Table 2: demographic data (continuous variables). Table 3: descriptive statistics (mean [SD]) of continuous demographic variables according to each trajectory group. Table 4: frequency counts (%) of categorical demographic variables according to each trajectory group. Figure 1: longitudinal trajectories of HbA1c values across adolescence (dash lines are 95% CIs); Group 1 accounts for 71.8% of the subjects and Group 2 accounts for 28.2% of the subjects. Supplement table 1: data extraction sheet and Supplement 2: detailed trajectory results.
