# Supplementary material for: Trajectory Analysis of Glycemic Control in Adolescents with Type 1 Diabetes Mellitus at Dammam Medical Complex, Saudi Arabia
Source: Adv Med. 2020 Dec 22;2020:1247294. doi: 10.1155/2020/1247294 (PMC7803114; doi:10.1155/2020/1247294)
Supplement: Supplementary Materials — Table 1: patient demographic data (categorical variables). Table 2: demographic data (continuous variables). Table 3: descriptive statistics (mean [SD]) of continuous demographic variables according to each trajectory group. Table 4: frequency counts (%) of categorical demographic variables according to each trajectory group. Figure 1: longitudinal trajectories of HbA1c values across adolescence (dash lines are 95% CIs); Group 1 accounts for 71.8% of the subjects and Group 2 accounts for 28.2% of the subjects. Supplement table 1: data extraction sheet and Supplement 2: detailed trajectory results. [file 1247294.f1.zip › 1247294.f1/mat.1247294.v1.docx]

**1. Analysis methods**

Latent class growth modelling (LCGM) (Nagin, 2005) was used to identify distinct subgroups of individuals following a distinct pattern of change over time on glycemic control (HbA1c). Latent class growth modeling provides semi-parametric statistics for distinguishing the developmental trajectories of subpopulations in sets (Nagin, 2005). Comparing to the standard growth curve modeling, where all individuals in a given sample are expected to change in the same direction across time with only the degree of change varying between people, the latent class growth modelling utilizes a group-based approach to provide statistics for distinguishing among clusters of trajectories within a population (Nagin, 2005; Andruff et al., 2009; Nagin, 2005).

The SAS procedure PROC TRAG developed by Jones, Nagin, and Roeder (2001) was implemented in SAS version 9.4 (SAS Inc., Cary, NC) to identify trajectories of glycemic control. Specifically, outcomes are treated as censored normal data following a polynomial

time course, given a discrete latent class assignment. In other words, one assumes disease histories (in this case, HbA1c trajectories) of I (i = 1, ..., N) subjects at T (t = 1, ..., T) times, and there are J (j = 1, ..., J) latent clusters of different histories in the population. Y_i_ = {y_i1_, ..., y_iT_} describes the longitudinal outcome measures of subject i over T times. Each cluster (i.e., trajectory) j is modeled by

$y_{it|j}^{*}=\beta_{0}^{j}+\beta_{1}^{j}{Age}_{it}+\beta_{2}^{j}{Age}_{it}^{2}+\beta_{3}^{j}{Age}_{it}^{3}+\varepsilon_{it}^{j}$ .

Note that

- ${Age}_{it}$ represents age of subject i at time t. Age of subject entered the model in a regular (linear, ${Age}_{it}$), squared (quadratic, ${Age}_{it}^{2}$), and cubic (${Age}_{it}^{3}$) term.
- $\beta_{0}^{j}$, $\beta_{1}^{j}$, $\beta_{2}^{j},$and $\beta_{3}^{j}$ represent parameters defining the intercept and slopes (i.e., linear, quadratic, and cubic) that determine the shape of the polynomial in cluster (trajectory) j;
- $\varepsilon_{it}^{j}$ is the normally distributed error term with zero mean and a constant standard deviation.

According to Nagin (2005), parameters of the LCGM are estimated by maximum likelihood and result in functions, where each function corresponds to a distinct trajectory and can be used to calculate probability of membership in each latent class for each subject. Note the change pattern of the trajectory could follow a linear trend (linear trend may either steadily increase or decrease at varying magnitudes or remain stable), a quadratic trend (a quadratic trend may increase, decrease, or remain stable up to a certain time point before changing in either magnitude or direction, or a cubic trend (a cubic trend will have two changes in either the magnitude or direction across time points) (Andruff et al., 2009).

The aim of the LCGM is to select the model with optimal number of distinct patterns as well as the appropriate polynomial order that represents the heterogeneity in trajectories (Nagin, 2005). The procedures established by Nagin (2005) and implemented by several researchers (Andruff et al., 2009; Helgeson et al., 2010; King et al., 2012; Baumgartner & Leydesdorff, 2014; Schwandt et al., 2017) for identifying the number of groups representing relatively homogenous clusters of trajectories of HbA1c over the study period of time, including 1) Determining the number of groups, 2) Selecting the shape of the pattern of change for each group over time, and 3) Determining model adequacy for the final model, were adopted in this study.

Step 1: Determining the number of groups

First, the raw data were examined graphically by plotting each individual’s HbA1c against age and examining the individual trajectories for distinct patterns of change. Next, to decide on the optimum number of groups for the data, a forward approach was undertaken, which

started with a one-class solution and then adds further classes. In other words, a one-group model, a two-group model, a three-group model, a four-group model, and a five-group model, with all groups set to a third order (cubic) equation as suggested by Nagin (2005), were fitted. The number of groups were determined by the Bayes information criterion (BIC). The BIC is the log-likelihood adjusted for the number of parameters and the sample size. In the PROC TRAJ procedure, the BIC values given in the output are negative, and hence the best fit model is the one with the largest negative number (i.e., smallest absolute value of BIC) (Nagin, 2005; Andruff et al., 2009; Baumgartner and Leydesdorff, 2014). Additionally, as BIC does not always explicitly identify an optimal number of groups, the context of the study objectives and also clinical relevance should be considered (Andruff et al., 2009; Schwandt et al., 2017).

Step 2: Selecting the shape of the pattern of change for each group over time

After identifying the number of groups, different shapes for the trajectories (i.e., linear, quadratic, and cubic) can be tested in the second step (Baumgartner and Leydesdorff, 2014; King et al., 2012). The final model was selected by examining the significance of the parameters and by comparing each model’s Bayesian Information Criterion value.

Step 3. Determining model adequacy

Once the ideal number of groups and shapes has been identified, in the third step, model adequacy can be tested using the average posterior probabilities (APP) of group membership (Nagin, 2005; Andruff et al., 2009; King et al., 2012; Baumgartner and Leydesdorff, 2014). The average posterior probabilities can be calculated by averaging the posterior probabilities of individuals having been assigned group membership to a trajectory using the maximum probability assignment rule. The average posterior probability of group membership for a trajectory ranges from 0 to 1 and is an approximation of the internal reliability for each trajectory. Average posterior probabilities of group membership greater than .70 indicate that the modelled trajectories accurately group individuals with similar patterns of change and discriminate between individuals with dissimilar patterns of change (Nagin, 2005). A further criterion discussed in literature for model accuracy is that each trajectory should include at least 5% of all subjects (Andruff et al., 2009; Twisk & Hoekstra, 2012).

After selecting the best model based on indicators of model fit and classification accuracy (a two-group model was identified), two-samples t-tests and Fisher’s exact tests were conducted to determine if there was a statistically significant difference in demographic and clinical variables, including

- initial age (i.e., age at the time study began),
- age at first diagnosis of T1DM,
- initial BMI (i.e., first BMI record for each subject in this study, a continuous variable),
- initial HbA1c (i.e., first HbA1c record for each subject in this study, a continuous variable),
- type of patient (a categorical variable with two levels: ER (patients newly diagnosed) vs. MCH (patients referred from pediatrics clinic)),
- initial insulin delivery method (a categorical variable with two levels: MID vs. MIX),
- gender (a categorical variable with two levels: male vs. female),
- average number of office visit (a continuous variable),
- DKA (a categorical variable with two levels: yes (DKA occurred at least once over the study period of time) vs. no (DKA did not occur)),
- hypothyroidism (a categorical variable with two levels: yes (hypothyroidism occurred at least once over the study period of time) vs. no (hypothyroidism did not occur)), and
- dyslipidemia (a categorical variable with two levels: yes (dyslipidemia occurred at least once over the study period of time) vs. no (dyslipidemia did not occur)),

between the two groups identified. Finally, a logistic regression was conducted to determine if any of the demographic and clinical variables can predict the trajectories. Results are given as odds ratios (OR) with 95% confidence intervals (CI). For any analyses, a p-value less than 0.05 indicates significance.

**2. Analysis results**

**Demographics**

Data of 44 T1DM patients were collected between 2006 and 2019 and analyzed in this study. Over the course of the years, starting as young as 11 years-old and ending as old as 19 years-old, patients were observed 4-8 times, which resulted in a total number of 272 observations for the study. Tables 1 and 2 presents the baseline demographics of the patients participated in this study. Among the 44 patients of this study, nearly two-thirds (61.36%) were referred from pediatrics clinics. All patients were Saudi Arabians (100%) and over half of the patients were female (54.55%). Most of the patients had started with separate multiple daily injection (MDI, 84.09%). For comorbidity, a considerable amount of patients had DKA (34.09%), dyslipidemia (20.45%), hypothyroidism (6.82%), and ingrowing toenail (6.82%), and less than 3% of the patients (2.27%) had mastoiditis, retinopathy, SCT, and chronic anterior uveitis.

The average age, BMI, and HbA1c at the time study began was 13.48 (SD = 1.34), 21.69 (4.00), and 11.54 (2.32). The average number of office visit over the course of the study was 1.79 (SD = 0.38). The average age at first diagnosis of T1DM was 10.11 (3.17).

Table 1: Demographics (categorical variables)

| Variable |  | N (%) |
| --- | --- | --- |
| Type of patient | ER (patients newly diagnosed) | 17 (38.64) |
|  | MCH (patients referred from pediatrics clinic) | 27 (61.36) |
| Initial insulin | MDI (separate multiple daily injection) | 37 (84.09) |
|  | MIX (mixed forms) | 7 (15.91) |
| Gender | Female | 24 (54.55) |
|  | Male | 20 (45.45) |
| Nationality | Saudi | 44 (100.00) |
| DKA | No | 29 (65.91) |
|  | Yes | 15 (34.09) |
| Hypothyroidism | No | 41 (93.18) |
|  | Yes | 3 (6.82) |
| Dyslipidemia | No | 35 (79.55) |
|  | Yes | 9 (20.45) |
| Mastoiditis | No | 43 (97.73) |
|  | Yes | 1 (2.27) |
| Ingrowing toenail | No | 43 (97.73) |
|  | Yes | 1 (2.27) |
| Retinopathy | No | 43 (97.73) |
|  | Yes | 1 (2.27) |
| SCT | No | 41 (93.18) |
|  | Yes | 3 (6.82) |
| Chronic anterior uveitis | No | 43 (97.73) |
|  | Yes | 1 (2.27) |

Note: For comorbidity, such as DKA, hypothyroidism, dyslipidemia, mastoiditis, ingrowing toenail, retinopathy, SCT, and chronic anterior uveitis, “Yes” indicates the condition occurred at least once during the study period and “No” indicates the condition did not occur during the study period.

Table 2: Demographics (continuous variables)

|  | Mean | SD | Min | Max |
| --- | --- | --- | --- | --- |
| Initial age | 13.48 | 1.34 | 11 | 16 |
| Initial BMI | 21.69 | 4.00 | 15.70 | 33.20 |
| Initial HbA1c | 11.54 | 2.32 | 5.90 | 16.00 |
| Average number of office visit | 1.79 | 0.38 | 1.20 | 2.80 |
| age at first diagnosis of T1DM | 10.11 | 3.17 | 1 | 15 |

Note: N = 44. N = 43 for initial BMI.

**Trajectory analysis**

To decide on the optimum number of groups for the data, the plot of individual’s HbA1c against age (Figure 1) and BIC of one-group, two-group, three-group, four-group, and five-group models with all groups set to a third order (cubic) equation (Table 3) were examined. Based on the visual inspection (Figure 1), two potential groups could be considered: a group with lower (10-12) and decreasing HbA1c, and a group with higher (12-16) and decreasing HbA1c. The BICs of one-group, two-group, three-group, four-group, and five-group models with all groups set to a third order (cubic) equation were -591.61, -567.89, -573.86, -575.79, and -581.52, respectively (Table 3). The two-group solution with all shapes defined as cubic therefore provided the best fit (BIC = -567.89). Additionally, previous studies had identified 5 groups with sample sizes 132 (Helgeson et al., 2017) and 6433 (Schwandt et al., 2017), 3 groups with sample sizes 222 (Rohan et al., 2015) and 155 (Hilliard et al., 2015), and 2 groups with sample sizes 132 (Helgeson et al., 2010) and 252 (King et al., 2012). Considering the sample size is small and the evidence of visual inspection of the data and the results of model selection based on BIC, the 2-group solution was chosen for this study.


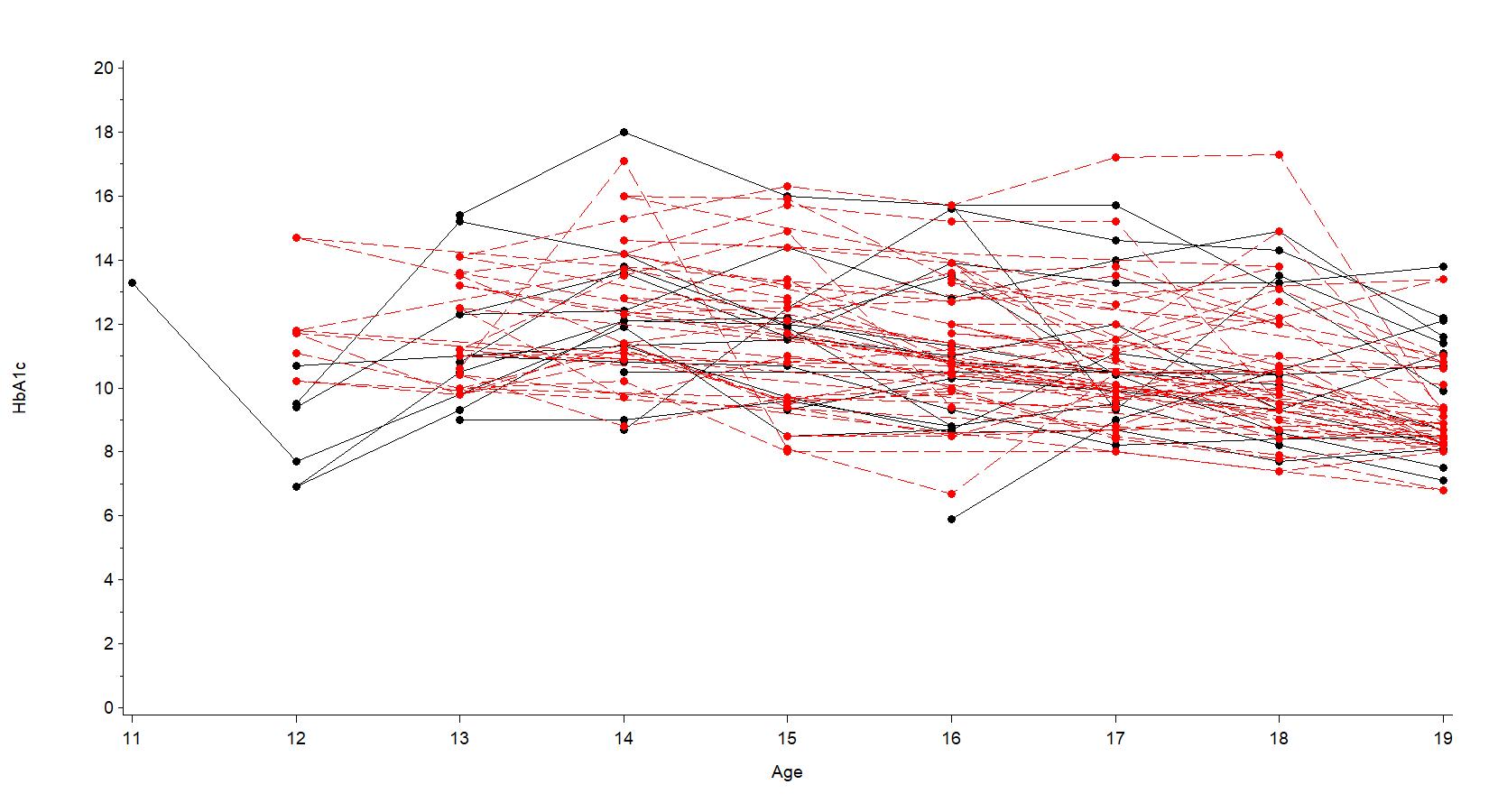


Figure 1: Plot of HbA1c against age (black line = ER, red line = MCH)

Table 3: BIC of one-group, two-group, three-group, four-group, and five-group models

| Model | BIC |
| --- | --- |
| One-group | -591.61 |
| Two-group | -567.89 |
| Three-group | -573.86 |
| Four-group | -575.79 |
| Five-group | -581.52 |

After identifying the number of groups, different shapes for the trajectories (i.e., linear, quadratic, and cubic) were examined by fitting polynomial functions with linear, quadratic and cubic orders, and the results are summarized in Table 4. Based on the BIC criterion and the significance of the parameters, the model with two trajectory groups (order(1, 2) with the smallest BIC and all significant parameters), the first of which was a linear group (intercept = 15.44, SE = 1.02, p < 0.0001; linear slope = -0.32, SE = 0.06, p < 0.0001) and the second of which was a quadratic group (intercept = -49.77, SE = 16.54, p = 0.0029; linear slope = 8.36, SE = 2.07, p < 0.0001; quadratic slope = -0.27, SE = 0.08, p < 0.0001) was selected.

In the two-class model, the first latent class (Group 1) contained 71.76% of the sample (n = 32). Group 1 had moderate HbA1c values at age 13 (HbA1c = 15.44-0.32*13 = 11.28) and had a significant and stable decrease in HbA1c with age (slope = -0.32, p < 0.0001). The second latent class (Group 2) contained 28.24% of the sample (n = 12). Group 2 demonstrated poor HbA1c values at age 13 (HbA1c = -49.77+8.36*13-0.27*13*13 = 13.28) and increased slightly through age 15 in HbA1c, and then stably decreased in HbA1c with age thereafter (linear slope = 8.36, quadratic slope = -0.27). Growth trajectories are depicted in Figure 2. The average posterior probability of group membership was 0.975 indicating that the modelled trajectories accurately grouped individuals with similar patterns of change and discriminated between individuals with dissimilar patterns of change.

Table 4: BIC and parameter estimates of the polynomial functions for the two trajectories

|  |  | Group 1 | | | | Group 2 | | | |
| --- | --- | --- | --- | --- | --- | --- | --- | --- | --- |
| Order | BIC | Intercept | linear | quadratic | cubic | Intercept | linear | quadratic | cubic |
| (1,1) | -563.49 | 15.31* | -0.31* | na | na | 19.45* | -0.39* | na | na |
| (1,2) | -555.98 | 15.44* | -0.32* | na | na | -49.77* | 8.36* | -0.27* | na |
| (1,3) | -557.85 | 15.43* | -0.32* | na | na | -22.48 | 3.13 | 0.06 | -0.01 |
| (2,1) | -563.65 | 0.68 | 1.58 | -0.06 | na | 19.07* | -0.36* | na | na |
| (2,2) | -556.39 | 2.78 | 1.33 | -0.05 | na | -49.69* | 8.35* | -0.27* | na |
| (2,3) | -558.24 | 2.65 | 1.34 | -0.05 | na | -11.11 | 0.95 | 0.20 | -0.01 |
| (3,1) | -564.37 | -82.73 | 18.07 | -1.13 | 0.02 | 19.03* | -0.36* | na | na |
| (3,2) | -557.15 | -74.80 | 16.67 | -1.05 | 0.02 | -49.15* | 8.28* | -0.27* | na |
| (3,3) | -558.99 | -75.43 | 16.78 | -1.06 | 0.02 | -5.12 | -0.15 | 0.26 | -0.01 |

Note: Polynomial order 1 = linear, 2 = quadratic, and 3 = cubic. For example, order (1, 2) indicates Group 1 (trajectory 1) was modeled using a linear equation and Group 2 (trajectory 2) was modeled using a quadratic equation. * indicates significance at the 0.05 level. na = not applicable.


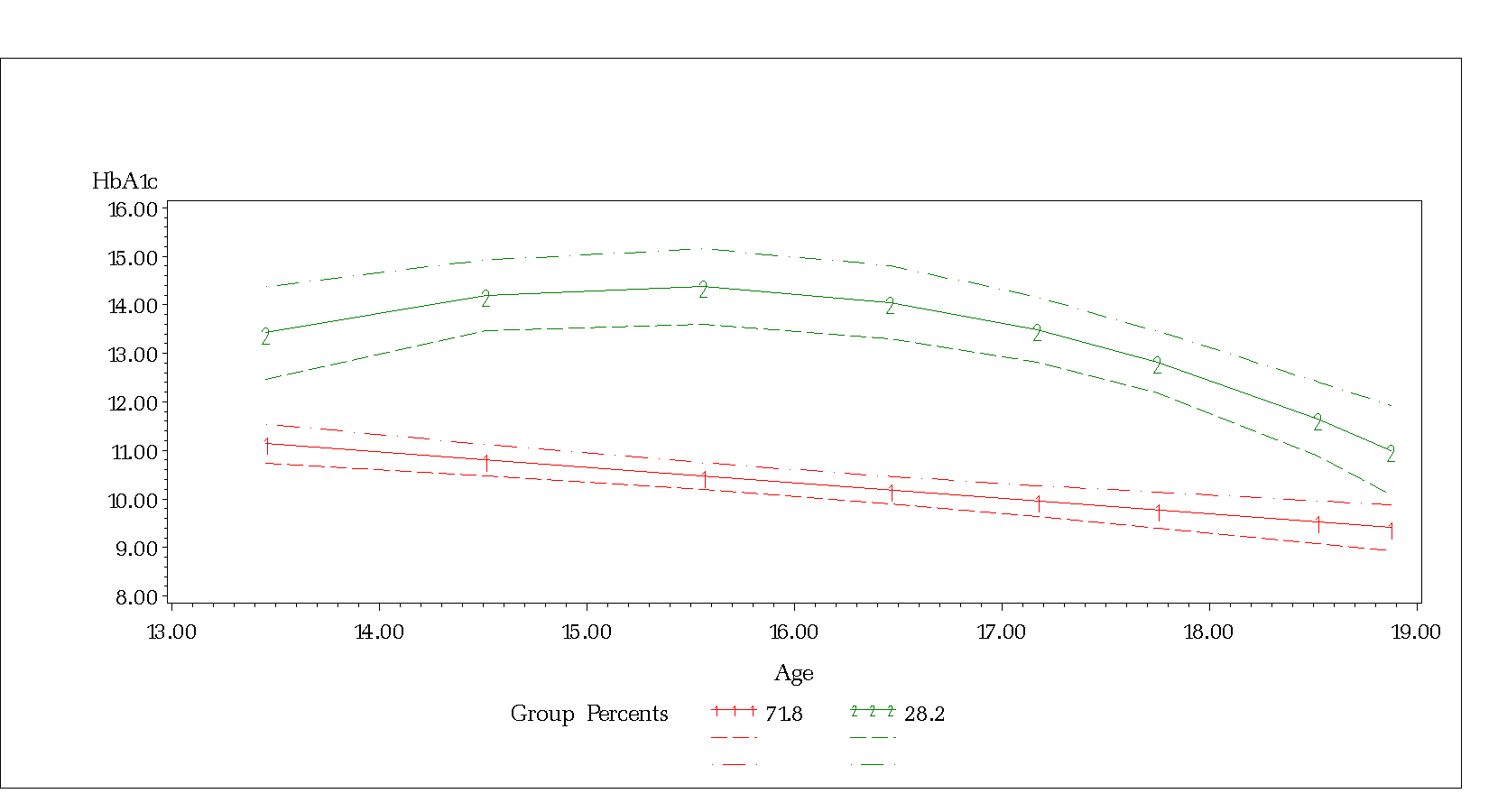


Figure 2: Longitudinal trajectories of HbA1c across adolescence (dash lines are 95% CIs), Group 1 accounts for 71.8% of the subjects and Group 2 accounts for 28.2% of the subjects.

**Demographics and trajectories**

Tables 5 and 6 presents the demographic characteristics by group of trajectories. According to two-sample t-tests, the initial BMI was statistically significantly higher for subjects in Group 1 than for subjects in Group 2 (M = 22.64, SD = 3.96 for Group 1; M = 19.25, SD = 3.05 for Group 2; t(41) = 2.66, p = 0.0110). The initial HbA1c was statistically significantly higher for subjects in Group 2 than for subjects in Group 1 (M = 10.90, SD = 1.98 for Group 1; M = 13.23, SD = 2.37 for Group 2; t(42) = -3.30, p = 0.0020). There was no statistically significant difference in initial age (t(42) = -1.62, p = 0.1135), average number of office visit (t(42) = -1.67, p = 0.1025), and age at first diagnosis of T1DM (t(42) = -0.81, p = 0.4214), between Group 1 and Group 2.

According to the results of Fisher’s exact tests, there was no statistically significant association between group of trajectories and type of patient (p = 1.0000), insulin insulin delivery method (p = 0.3694), gender (p = 0.7456), DKA (p = 1.0000), hypothyroidism (p = 1.0000), and dyslipidemia (p = 0.0868).

Table 5: Descriptive statistics (Mean (SD)) of continuous demographic variables by group of trajectories

|  | Group 1 | Group 2 | t | df | p |
| --- | --- | --- | --- | --- | --- |
| Initial age | 13.28 (1.35) | 14.00 (1.21) | -1.62 | 42 | 0.1135 |
| Initial BMI | 22.64 (3.96) | 19.25 (3.05) | 2.66 | 41 | 0.0110 |
| Initial HbA1c | 10.90 (1.98) | 13.23 (2.37) | -3.30 | 42 | 0.0020 |
| Average number of office visit | 1.84 (0.41) | 1.63 (0.23) | 1.67 | 42 | 0.1025 |
| age at first diagnosis of T1DM | 9.88 (3.35) | 10.75 (2.67) | -0.81 | 42 | 0.4214 |

Table 6: Frequency counts (%) of categorical demographic variables by group of trajectories

|  |  | Group 1 | Group 2 | p |
| --- | --- | --- | --- | --- |
| Type of patient | ER | 12 (37.50) | 5 (41.67) | 1.0000 |
|  | MCH | 20 (62.50) | 7 (58.33) |  |
| Insulin insulin delivery | MDI | 28 (87.50) | 9 (75.00) | 0.3694 |
|  | MIX | 4 (12.50) | 3 (25.00) |  |
| Gender | Female | 18 (56.25) | 6 (50.00) | 0.7456 |
|  | Male | 14 (43.75) | 6 (50.00) |  |
| DKA | No | 21 (65.63) | 8 (66.67) | 1.0000 |
|  | Yes | 11 (34.38) | 4 (33.33) |  |
| Hypothyroidism | No | 30 (93.75) | 11 (91.67) | 1.0000 |
|  | Yes | 2 (6.25) | 1 (8.33) |  |
| Dyslipidemia | No | 29 (87.50) | 7 (58.33) | 0.0868 |
|  | Yes | 4 (12.50) | 5 (41.67) |  |

Note: For comorbidity, such as DKA, hypothyroidism, and dyslipidemia, “Yes” indicates the condition occurred at least once during the study period and “No” indicates the condition did not occur during the study period.

**Prediction of trajectories**

A logistic regression was conducted to determine if any of the demographic and clinical variables can predict the trajectories (Table 7). The results indicated that initial HbA1c was a significant predictor for group trajectory (χ^2^(1) = 5.8762, p = 0.0153). In particular, subjects with higher initial HbA1c were 98.47% less likely to be in Group 1 (OR = 0.0153, 95% CI = [0.311, 0.884]). The remaining variables, including type of patient (χ^2^(1) = 1.8535, p = 0.1769), initial insulin delivery method (χ^2^(1) = 0.9270, p = 0.3356), gender (χ^2^(1) = 0.1147, p = 0.7348), DKA (χ^2^(1) = 0.1041, p = 0.7470), hypothyroidism (χ^2^(1) = 0.0805, p = 0.7766), dyslipidemia (χ^2^(1) = 2.2281, p = 0.1355), initial age (χ^2^(1) = 1.0440, p = 0.3069), initial BMI (χ^2^(1) = 2.4054, p = 0.1203), average number of office visits (χ^2^(1) = 0.7672, p = 0.3811), and age of first diagnosis of T1DM (χ^2^(1) = 0.3340, p = 0.5628), were not significant predictors for group trajectory. Hosmer and Lemeshow goodness-of-fit test indicated that the model fit was adequate (χ^2^(9) = 6.2614, p = 0.7135).

Table 7: Results of logistic regression

| Variable | Level | Df | Wald chi-square test | p | OR | 95% CI of OR |
| --- | --- | --- | --- | --- | --- | --- |
| Type of patient | ER | 1 | 1.8535 | 0.1769 | 0.209 | [0.021, 2.028] |
|  | MCH |  |  |  | Ref |  |
| Initial insulin delivery method | MDI | 1 | 0.9270 | 0.3356 | 3.146 | [0.305, 32.430] |
|  | MIX |  |  |  | Ref |  |
| Gender | Female | 1 | 0.1147 | 0.7348 | 1.308 | [0.277, 6.185] |
|  | Male |  |  |  | Ref |  |
| DKA | No | 1 | 0.1041 | 0.7470 | 0.716 | [0.094, 5.465] |
|  | Yes |  |  |  | Ref |  |
| Hypothyroidism | No | 1 | 0.0805 | 0.7766 | 1.803 | [0.031, 105.860] |
|  | Yes |  |  |  | Ref |  |
| Dyslipidemia | No | 1 | 2.2281 | 0.1355 | 7.090 | [0.542, 92.500] |
|  | Yes |  |  |  | Ref |  |
| Initial age |  | 1 | 1.0440 | 0.3069 | 0.698 | [0.351, 1.391] |
| Initial BMI |  | 1 | 2.4054 | 0.1203 | 1.190 | [0.955, 1.482] |
| Initial HbA1c |  | 1 | 5.8762 | 0.0153 | 0.524 | [0.311, 0.884] |
| Average number of office visits |  | 1 | 0.7672 | 0.3811 | 3.108 | [0.246, 39.317] |
| Age of first diagnosis of T1DM |  | 1 | 0.3340 | 0.5628 | 0.921 | [0.697, 1.217] |

Note: The logistic regression modeled probability of being Group 1. Ref = reference group.

**References**

Andruff, H., Carraro, N., Thompson, A., and GaudreauLatent, P. (2009). Class Growth Modelling: A Tutorial. Tutorials in Quantitative Methods for Psychology, 5, 1, 11-24.

Baumgartner, S. E., & Leydesdorff, L. (2014). Group‐based trajectory modeling (GBTM) of citations in scholarly literature: dynamic qualities of “transient” and “sticky knowledge claims”. Journal of the Association for Information Science and Technology, 65(4), 797-811.

Helgeson, V. S., Snyder, P. R., Seltman, H., Escobar, O., Becker, D., & Siminerio, L. (2010). Brief report: trajectories of glycemic control over early to middle adolescence. Journal of Pediatric Psychology, 35(10), 1161-1167.

Helgeson, V. S., Vaughn, A. K., Seltman, H., Orchard, T., Libman, I., & Becker, D. (2017). Trajectories of glycemic control over adolescence and emerging adulthood: an 11-year longitudinal study of youth with type 1 diabetes. *Journal of pediatric psychology*, *43*(1), 8-18.

Hilliard, M. E., Wu, Y. P., Rausch, J., Dolan, L. M., & Hood, K. K. (2013). Predictors of deteriorations in diabetes management and control in adolescents with type 1 diabetes. *Journal of Adolescent Health*, *52*(1), 28-34.

Jones, B. L., Nagin, D. S., & Roeder, K. (2001). A SAS procedure based on mixture models for estimating developmental trajectories. *Sociological methods & research*, *29*(3), 374-393.

King, P.S., Berg, C.A., Butner, J., Drew, L.M., Foster, C., Donaldson, D., Murray, M., Swinyard, M. and Wiebe, D.J. (2012). Longitudinal trajectories of metabolic control across adolescence: Associations with parental involvement, adolescents' psychosocial maturity, and health care utilization. Journal of Adolescent Health, 50(5), 491-496.

Nagin, D. S. (2005). Group-based Modeling of Development. Massachusetts: Harvard University Press.

Rohan, J. M., Rausch, J. R., Pendley, J. S., Delamater, A. M., Dolan, L., Reeves, G., & Drotar, D. (2014). Identification and prediction of group-based glycemic control trajectories during the transition to adolescence. *Health Psychology*, *33*(10), 1143.

Schwandt, A., Hermann, J.M., Rosenbauer, J., Boettcher, C., Dunstheimer, D., Grulich-Henn, J., Kuss, O., Rami-Merhar, B., Vogel, C. and Holl, R.W. (2017). Longitudinal trajectories of metabolic control from childhood to young adulthood in type 1 diabetes from a large German/Austrian registry: a group-based modeling approach. Diabetes Care, 40(3), 309-316.

Twisk, J., & Hoekstra, T. (2012). Classifying developmental trajectories over time should be done with great caution: a comparison between methods. *Journal of clinical epidemiology*, *65*(10), 1078-1087.
