# Supplementary material for: Trajectory Analysis of Glycemic Control in Adolescents with Type 1 Diabetes Mellitus at Dammam Medical Complex, Saudi Arabia
Source: Adv Med. 2020 Dec 22;2020:1247294. doi: 10.1155/2020/1247294 (PMC7803114; doi:10.1155/2020/1247294)
Supplement: Supplementary Materials — Table 1: patient demographic data (categorical variables). Table 2: demographic data (continuous variables). Table 3: descriptive statistics (mean [SD]) of continuous demographic variables according to each trajectory group. Table 4: frequency counts (%) of categorical demographic variables according to each trajectory group. Figure 1: longitudinal trajectories of HbA1c values across adolescence (dash lines are 95% CIs); Group 1 accounts for 71.8% of the subjects and Group 2 accounts for 28.2% of the subjects. Supplement table 1: data extraction sheet and Supplement 2: detailed trajectory results. [file 1247294.f1.zip › 1247294.f1/supplement table 1.docx]

| File number | Age | sex | Year of first encounter | Nationality | Duration of diabetes |
| --- | --- | --- | --- | --- | --- |
|  |  |  |  |  |  |

**Supplement table 1**

| Comorbidities | Present or absent | year |
| --- | --- | --- |
| Retinopathy (documented in the file by ophthalmologist) |  |  |
| Nephropathy (Cr clearance less than 90 and /or presence of moderately or severely increased albuminuria in two 24hr urine sample |  |  |
| Neuropathy (documented by neurologist or on treatment ) |  |  |
| Dyslipidemia (LDL more than 130 , TG more than 150 or been on treatment) |  |  |
| Celiac disease (documented by gastroenterologist ) |  |  |
| Hypertension (on anti-HTN drugs ) |  |  |
| Hypothyroidism (on L-thyroxin) |  |  |
| Subclinical hypothyroidism (TSH>5, normal FT4 in two occasions ) |  |  |
| Haemoglobinopthy (HBG electrophoresis ,follow up with hematology ) |  |  |
| Others |  |  |

**Data extraction sheet:**

| Visit date | HBa1c | BMI | Insulin delivery method( insulin pump, multiple daily injection, mixed insulin) |
| --- | --- | --- | --- |
|  |  |  |  |
|  |  |  |  |
|  |  |  |  |
|  |  |  |  |
|  |  |  |  |
|  |  |  |  |
|  |  |  |  |
|  |  |  |  |

| Admission history | Date |
| --- | --- |
| Diabetic ketoacidosis |  |
| Hypoglycemia |  |
| Diabetic foot |  |
| Other surgical or medical causes |  |
