# Supplementary material for: Trajectory Analysis of Glycemic Control in Adolescents with Type 1 Diabetes Mellitus at Dammam Medical Complex, Saudi Arabia
Source: Adv Med. 2020 Dec 22;2020:1247294. doi: 10.1155/2020/1247294 (PMC7803114; doi:10.1155/2020/1247294)
Supplement: Supplementary Materials — Table 1: patient demographic data (categorical variables). Table 2: demographic data (continuous variables). Table 3: descriptive statistics (mean [SD]) of continuous demographic variables according to each trajectory group. Table 4: frequency counts (%) of categorical demographic variables according to each trajectory group. Figure 1: longitudinal trajectories of HbA1c values across adolescence (dash lines are 95% CIs); Group 1 accounts for 71.8% of the subjects and Group 2 accounts for 28.2% of the subjects. Supplement table 1: data extraction sheet and Supplement 2: detailed trajectory results. [file 1247294.f1.zip › 1247294.f1/Table 1.docx]

**Table 1.** Patient demographic data (categorical variables)

| Variable |  | N (%) |
| --- | --- | --- |
| Patient type | ED (patients newly diagnosed in emergency department ) | 17 (38.6) |
|  | MCH (patients referred from the pediatrics clinic) | 27 (61.3) |
| Initial insulin | separate multiple daily injection (MDI) | 37 (84.0) |
|  | mixed insulin forms twice daily (MIX) | 7 (15.9) |
| Gender | Female | 24 (54.5) |
|  | Male | 20 (45.4) |
| Nationality | Saudi | 44 (100) |
| DKA | No* | 29 (65.9) |
|  | Yes* | 15 (34.0) |
| Hypothyroidism | No | 41 (93.1) |
|  | Yes | 3 (6.8) |
| Dyslipidemia | No | 35 (79.5) |
|  | Yes | 9 (20.4) |
| Retinopathy | No | 43 (97.7) |
|  | Yes | 1 (2.2) |
| SCT | No | 41 (93.1) |
|  | Yes | 3 (6.8) |

* ‘Yes’ indicates the condition had occurred at least once during the study period and ‘No’ indicates the condition had not occur during the study period.

DKA, diabetic ketoacidosis, ED, emergency department; MCH, Maternity and Children’s Hospital; SCT, Sickle Cell Trait.
