# Supplementary material for: Trajectory Analysis of Glycemic Control in Adolescents with Type 1 Diabetes Mellitus at Dammam Medical Complex, Saudi Arabia
Source: Adv Med. 2020 Dec 22;2020:1247294. doi: 10.1155/2020/1247294 (PMC7803114; doi:10.1155/2020/1247294)
Supplement: Supplementary Materials — Table 1: patient demographic data (categorical variables). Table 2: demographic data (continuous variables). Table 3: descriptive statistics (mean [SD]) of continuous demographic variables according to each trajectory group. Table 4: frequency counts (%) of categorical demographic variables according to each trajectory group. Figure 1: longitudinal trajectories of HbA1c values across adolescence (dash lines are 95% CIs); Group 1 accounts for 71.8% of the subjects and Group 2 accounts for 28.2% of the subjects. Supplement table 1: data extraction sheet and Supplement 2: detailed trajectory results. [file 1247294.f1.zip › 1247294.f1/Table 2.docx]

**Table 2**. Demographic data (continuous variables)

|  | Mean | SD | Min | Max |
| --- | --- | --- | --- | --- |
| Initial age at the clinic | 13.4 | 1.3 | 11 | 16 |
| Initial BMI | 21.6 | 4.0 | 15.7 | 33.2 |
| Initial HbA1c | 11.5 | 2.3 | 5.9 | 16.0 |
| Average number of office visit | 1.7 | 0.3 | 1.2 | 2.8 |
| Age at first diagnosis of T1DM | 10.1 | 3.1 | 1 | 15 |

Note: N = 44.

Abbreviations: BMI, body mass index; HbA1c, glycated hemoglobin (%); SD, standard deviation; T1DM, type 1 diabetes mellitus
