# Supplementary material for: Trajectory Analysis of Glycemic Control in Adolescents with Type 1 Diabetes Mellitus at Dammam Medical Complex, Saudi Arabia
Source: Adv Med. 2020 Dec 22;2020:1247294. doi: 10.1155/2020/1247294 (PMC7803114; doi:10.1155/2020/1247294)
Supplement: Supplementary Materials — Table 1: patient demographic data (categorical variables). Table 2: demographic data (continuous variables). Table 3: descriptive statistics (mean [SD]) of continuous demographic variables according to each trajectory group. Table 4: frequency counts (%) of categorical demographic variables according to each trajectory group. Figure 1: longitudinal trajectories of HbA1c values across adolescence (dash lines are 95% CIs); Group 1 accounts for 71.8% of the subjects and Group 2 accounts for 28.2% of the subjects. Supplement table 1: data extraction sheet and Supplement 2: detailed trajectory results. [file 1247294.f1.zip › 1247294.f1/Table 3.docx]

**Table 3**. Descriptive statistics (mean [SD]) of continuous demographic variables according to each trajectory group

|  | Group 1 | Group 2 | t | df | p |
| --- | --- | --- | --- | --- | --- |
| Initial age | 13.2 (1.3) | 14.0 (1.2) | -1.6 | 42 | 0.11 |
| Initial BMI | 22.6 (3.9) | 19.2 (3.0) | 2.6 | 41 | 0.01 |
| Initial HbA1c value | 10.9 (1.9) | 13.2 (2.3) | -3.3 | 42 | 0.00 |
| Average number of office visits | 1.8 (0.4) | 1.6 (0.2) | 1.6 | 42 | 0.10 |
| Age at first diagnosis of T1DM | 9.8 (3.3) | 10.7 (2.6) | -0.8 | 42 | 0.42 |

BMI, body mass index; HbA1c, glycated hemoglobin (%); T1DM, type 1 diabetes mellitus
