# Supplementary material for: Trajectory Analysis of Glycemic Control in Adolescents with Type 1 Diabetes Mellitus at Dammam Medical Complex, Saudi Arabia
Source: Adv Med. 2020 Dec 22;2020:1247294. doi: 10.1155/2020/1247294 (PMC7803114; doi:10.1155/2020/1247294)
Supplement: Supplementary Materials — Table 1: patient demographic data (categorical variables). Table 2: demographic data (continuous variables). Table 3: descriptive statistics (mean [SD]) of continuous demographic variables according to each trajectory group. Table 4: frequency counts (%) of categorical demographic variables according to each trajectory group. Figure 1: longitudinal trajectories of HbA1c values across adolescence (dash lines are 95% CIs); Group 1 accounts for 71.8% of the subjects and Group 2 accounts for 28.2% of the subjects. Supplement table 1: data extraction sheet and Supplement 2: detailed trajectory results. [file 1247294.f1.zip › 1247294.f1/Table 4.docx]

**Table 4**. Frequency counts (%) of categorical demographic variables according to each trajectory group

|  |  | Group 1 | Group 2 | p-value |
| --- | --- | --- | --- | --- |
| Patient type | ED | 12 (37.5) | 5 (41.6) | 1.0 |
|  | MCH | 20 (62.5) | 7 (58.3) |  |
| Insulin regimen | MDI | 28 (87.5) | 9 (75.0) | 0.3 |
|  | MIX | 4 (12.5) | 3 (25.0) |  |
| Gender | Female | 18 (56.2) | 6 (50.0) | 0.7 |
|  | Male | 14 (43.7) | 6 (50.0) |  |
| Diabetic ketoacidosis | No* | 21 (65.6) | 8 (66.6) | 1.0 |
|  | Yes* | 11 (34.3) | 4 (33.3) |  |
| Hypothyroidism | No | 30 (93.7) | 11 (91.6) | 1.0 |
|  | Yes | 2 (6.2) | 1 (8.3) |  |
| Dyslipidemia | No | 29 (87.5) | 7 (58.3) | 0.08 |
|  | Yes | 4 (12.5) | 5 (41.6) |  |

*For comorbidities such as diabetic ketoacidosis, hypothyroidism, and dyslipidemia, ‘Yes’ indicates the condition had occurred at least once during the study period. ‘No’ indicates the condition had not occurred during the study period.

ED, emergency department; MCH, Maternity and Children’s Hospital; MDI, Multiple Daily Injection; MIX, Twice daily premixed insulin.
